# Analysis of a Medication Safety Intervention in the Pediatric Emergency Department

**DOI:** 10.1001/jamanetworkopen.2023.51629

**Published:** 2024-01-12

**Authors:** Margaret E. Samuels-Kalow, Randall Tassone, William Manning, Rebecca Cash, Laura Davila-Parrilla, Bryan D. Hayes, Stephen Porter, Carlos A. Camargo

**Affiliations:** 1 Department of Emergency Medicine, Massachusetts General Hospital, Harvard Medical School, Boston; 2 Ponce Health Sciences University School of Medicine, Ponce, Puerto Rico; 3 Division of Emergency Medicine, Cincinnati Children’s Hospital Medical Center, Cincinnati, Ohio; 4 Department of Pediatrics, University of Cincinnati College of Medicine, Cincinnati, Ohio

## Abstract

**Importance:**

Strategies to reduce medication dosing errors are crucial for improving outcomes. The Medication Education for Dosing Safety (MEDS) intervention, consisting of a simplified handout, dosing syringe, dose demonstration and teach-back, was shown to be effective in the emergency department (ED), but optimal intervention strategies to move it into clinical practice remain to be described.

**Objective:**

To describe implementation of MEDS in routine clinical practice and associated outcomes.

**Design, Setting, and Participants:**

This mixed-methods interrupted time series study of MEDS was conducted April 2021 to December 2022 in an academic pediatric ED using a hybrid type 1 design. Parents and guardians of children aged 90 days to 11.9 years who were discharged with acetaminophen, ibuprofen, or both were eligible for inclusion in the quantitative portion. Clinicians from a diversity of role groups (attending physician, resident, and nurse) were eligible for the qualitative portion.

**Exposures:**

The study was conducted in 5 stages (baseline, intervention 1, washout, intervention 2, and sustainability phases). The 2 intervention phases taught clinical staff the MEDS intervention using different implementation strategies. During the intervention 1 phase, in-depth interviews were conducted until thematic saturation was reached; results were analyzed using thematic analysis. Interviews informed intervention 2 phase interventions.

**Main Outcomes and Measures:**

The primary outcome was any error (defined as dosing or frequency error) at a 48- to 72-hour follow-up phone call.

**Results:**

There were 256 participants (median [IQR] child age, 1.7 [3.0-7.0] years; median [IQR] parent and guardian age, 36.0 [31.0-41.0] years; 200 females among parents and guardians [78.1%]) who consented and completed follow-up. At baseline, 44 of 68 participants (64.7%) made an error compared with 34 of 65 participants (52.3%) during intervention 1, 31 of 63 participants (49.X%) during intervention 2, and 34 of 60 participants (57.X%) during sustainability. After adjustment for language and health literacy, the adjusted odds ratio for error during the combined intervention phases was 0.52 (95% CI, 0.28-0.97) compared with baseline.

**Conclusions and Relevance:**

This study found that both MEDS intervention phases were associated with decreased risk of error and that some improvement was sustained without active intervention. These findings suggest that attempts to develop simplified, brief interventions may be associated with improved medication safety for children after discharge from the ED.

## Key Points

**Question:**

What were the outcomes after implementation of the Medication Education for Dosing Safety (MEDS) in routine clinical practice?

**Findings:**

This mixed-methods study of 256 parents and guardians of children being discharged from the emergency department developed and tested implementation strategies for the MEDS intervention and found that they were associated with decreased risk of error and sustained improvement without active training.

**Meaning:**

These findings suggest that clinicians may be trained to deliver simple, brief interventions associated with improved dosing safety.

## Teaser

This mixed-methods study analyzed outcomes of the Medication Education for Dosing Safety in a pediatric emergency department.

## Introduction

Approximately 63 000 children yearly are affected by medication errors at home.^1^ Rates of medication dosing error range from 30% to 80%.^2^ Limited health literacy^3^ and limited English proficiency are common among families with children in the emergency department (ED), and both are associated with increased dosing error.^4,5,6,7^

Despite studies suggesting that standardized verbal instructions,^8^ assessing recall and comprehension of new information (teach-back),^9^ and provision of dosing syringes are associated with a decreased risk of error^10^ and improved outcomes, these strategies are not routinely used in ED discharge teaching. Current ED discharge instructions are brief,^11,12^ lack key components,^12,13^ and provide limited opportunities to confirm understanding,^14^ with low rates of checking for comprehension^14^ and dose demonstration.^12^ Patients and families often leave the ED with insufficient understanding,^15^ which frequently goes unrecognized.^16^ This results in high rates of dosing error; in 1 study,^12^ 32% of parents had an acetaminophen dosing error when assessed immediately after the child’s ED discharge, despite provision of a dosing instruction sheet.

The MEDS (Medication Education for Dosing Safety) intervention, in which parents and guardians are provided with a simplified dosing handout and syringe, dosing demonstration, and teach-back process, reduced the risk of error for families leaving the ED when delivered by a research assistant.^17^ The goal of this study was to examine outcomes associated with the MEDS intervention in routine clinical practice and to investigate factors associated with successful implementation of the intervention.

## Methods

### Ethics Approval and Reporting

This mixed-methods study was determined to be exempt from review by the Mass General Brigham Institutional Review Board because it met criteria for exemption per 45 CFR §46.104(d). Parents and guardians provided informed consent. The study is reported according to the Consolidated Criteria for Reporting Qualitative Research (COREQ) reporting guideline (eAppendix 2-3 in Supplement 1).

### Study Design and Setting

We conducted an interrupted time-series study in an academic pediatric ED April 2021 to December 2022 using a hybrid type 1 effectiveness-implementation design, testing the association of the intervention with outcomes while gathering data on implementation.^18^ Primary interventions were 2 forms of training for clinicians to implement the MEDS intervention. Overall, the study occurred in 5 phases (Figure). The first phase was a baseline observation phase, followed by an initial training phase (intervention 1), a washout period, a second training phase (intervention 2), and a sustainability phase in which the research team no longer provided active assistance for the intervention.

**Figure.**
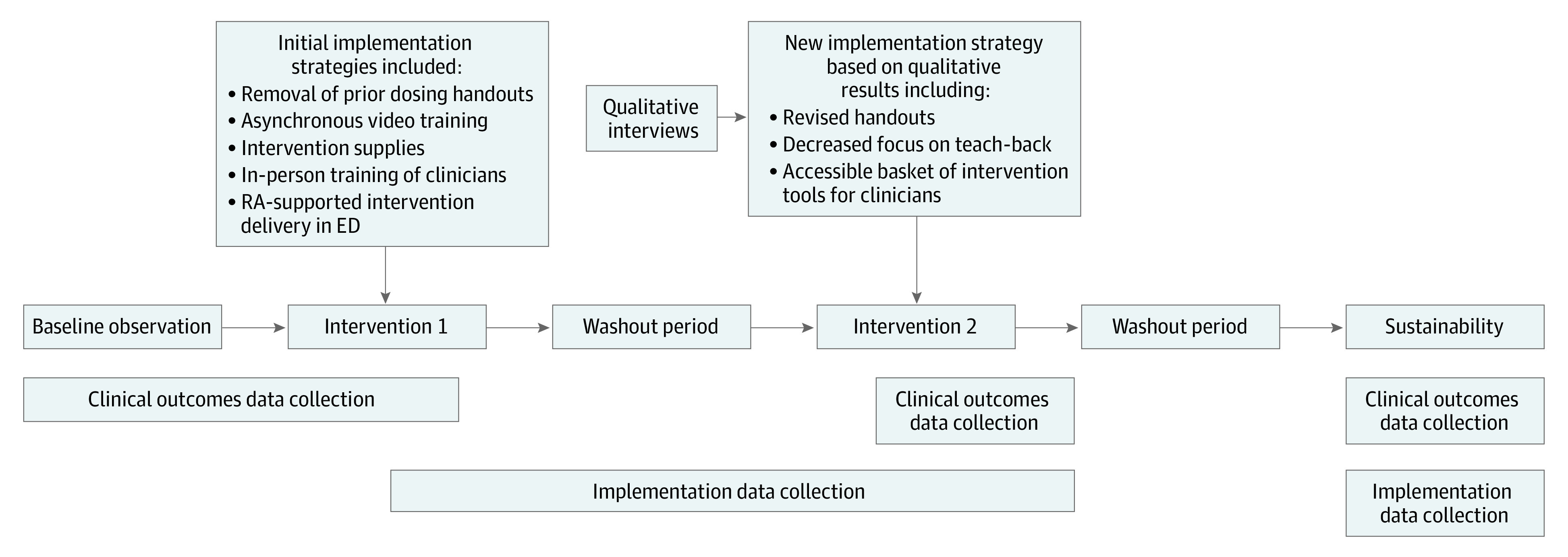
Overall Study Design ED indicates emergency department; RA, research assistant.

### Participants

#### Quantitative

Parents and guardians of children aged 90 days to 11.9 years who were discharged with acetaminophen, ibuprofen, or both were eligible. Inclusion criteria included parental fluency in English or Spanish, ability to be reached by telephone over the next 7 days, and planned discharge home. Exclusion criteria included the presence of a complex chronic condition^19^ in the child, planned use of a nonstandard medication dose, and not being accompanied by a guardian. Research assistant (RA) shifts were varied by day of week and time of day, and RAs attempted to enroll all eligible patients during a shift.

#### Qualitative

Interviews were conducted with selected clinicians from a diversity of role groups (attending, resident, and nurse) recruited by email. The sampling strategy was purposive, designed to balance across role groups.^20^

### Interventions by Phase

#### Baseline Phase

RAs enrolled and consented patients and collected data. The RA did not provide study materials to the clinical team.

#### Intervention 1

The initial training involved a video introduction to MEDS instructions, an in-person orientation, and 1-on-1, just-in-time training of available clinicians in the ED by RAs. This was offered to all residents working in the ED (including emergency medicine and pediatric EDs), nurses, and attending clinicians. Electronic versions of MEDS instructions were created and incorporated into the electronic health record for insertion into discharge instructions (after-visit summaries) in English and Spanish, and a folder with printed handouts was made available in the nursing workstation. Reminder stickers were placed on clinician computers as visual cues. RAs supported intervention delivery by assisting with identifying eligible families and accessing intervention materials for clinicians. During this period, RAs enrolled, consented, and collected data from patients.

#### Washout Period and Qualitative Data Collection and Analysis

During this period, no patients were enrolled. Qualitative interviews with clinicians focused on identifying strategies to ensure that the intervention could be delivered consistently over time and institutionalized.^21^ Insights from qualitative interviews were used to modify implementation strategies for use in the intervention 2 phase.

#### Intervention 2

Findings from qualitative interviews were synthesized using the Framework for Reporting Adaptations and Modifications-Expanded (FRAME).^22^ Modifications were included in the intervention 2 based on study team determination of feasibility and fit (see the FRAME table in eAppendix 1 in Supplement 1). Intervention 2 was offered to residents rotating in the ED during that time, nurses, and attending physicians; these individuals may or may not have seen the initial training. During this period, RAs enrolled, consented, and collected data from patients.

#### Sustainability Phase

After a second washout period of 8 weeks, active RA assistance was concluded. A new RA enrolled and consented patients and collected data to avoid any potential bias from staff recognizing prior RA association with the study.

### Outcomes and Measurement

#### Quantitative

After consent and enrollment, parents and guardians completed a survey collecting demographic information and the Newest Vital Sign (to assess health literacy and numeracy), which has been validated in English and Spanish.^23^ Race and ethnicity were self-reported by the parent or guardian using separate race (American Indian or Alaska Native, Asian, Black or African American, Native Hawaiian or Other Pacific Islander, White, other, and refuse) and ethnicity (Hispanic, yes or no) questions. For analysis, categories were combined as Hispanic (any race), non-Hispanic Black, non-Hispanic White, and non-Hispanic other, which includes participants whose ethnicity was not Hispanic and whose race was not White or Black. Self-reported race and ethnicity were assessed to provide baseline data regarding study populations, diversity of enrollment, and generalizability of the included sample. Safe dosing, assessed by phone interview at the 48- to 72-hour follow-up, was defined as dosing within a 20% range of the weight-based dose^10,24^ as written on the provided handout. Participants were asked to describe the correct dose and frequency for acetaminophen or ibuprofen for their child. Parents and guardians who were using a given medication were asked about the dose they had given. Parents and guardians who were not using the medication were asked to describe the hypothetical dose and frequency schedule for their child. All discharge processes were directly observed for implementation outcomes, which were drawn from the Proctor framework^25^ and included adoption, fidelity, and efficiency (time of process).

The primary outcome was any error (dosing or frequency error of either medication, whether it was being used or in hypothetical dosing questions). As secondary outcomes, we examined actual error (dosing or frequency errors of a medication the family actually used) and potential use error (dosing or frequency errors of a medication the family used or was told to use by the clinical team). We also separately examined risks of underdosing and overdosing errors.

#### Qualitative

The qualitative examination of the clinician training and implementation process was based on the dynamic sustainability framework (DSF).^21^ The DSF emphasizes the importance of the fit among the intervention, practice setting, and ecological system, recognizing how this may change over time. All interviews were recorded and professionally transcribed.

### Sample Size

Each enrollment phase was conducted until a minimum of 65 participants were enrolled and completed follow-up activities. This threshold was used given that we required 61 participants per stage to meet our a priori power calculation based on the expected difference between baseline and sustainability periods, using the difference (45% vs 71%) found in our pilot study,^17^ for a power of 80% and an α of .05.

### Statistical Analysis

#### Quantitative

We used standard descriptive statistics for patient characteristics, the discharge process, and outcomes by study phase. Available case analysis was used to handle missing data. Comparisons by study phase were made using χ^2^ or Kruskal-Wallis tests, as appropriate. A 2-sided α = .05 was used as the level of statistical significance. Data were analyzed using Stata statistical software version 15.1 (StataCorp). We fit individual multivariable logistic regression models comparing intervention periods to the baseline for primary and secondary outcomes, adjusting for language and health literacy as potential confounders.

#### Qualitative

Coding and theme generation were ongoing, and thematic analysis was used.^26,27^ We used a deductive approach and the DSF framework.^28^ Interviews were completed until thematic saturation was identified by consensus, meaning that no new themes were being developed from subsequent interviews. A coding tree was developed based on the interview guide and then refined with input from the team. Transcripts were coded by 2 independent staff members (W.M. and L.D.P.), with differences resolved by consensus. Themes were generated and mapped to DSF elements.^21^

## Results

### Quantitative: Enrollment, Baseline, and Intervention 1

A total of 2424 patients were screened, of whom 503 individuals (20.8%) were eligible; parents and guardians for 326 eligible patients (64.8%) consented, and 256 individuals who consented (78.5%) completed the follow-up call (Table 1). Among these 256 participants (median [IQR] child age, 1.7 [3.0-7.0] years; median [IQR] parent or guardian age, 36.0 [31.0-41.0] years; 200 females among parents and guardians [78.1%]; 109 Hispanic or Latino [42.8%], 15 non-Hispanic Black [5.9%], 101 non-Hispanic White [40.0%], and 30 non-Hispanic other race [11.8%] among parents and guardians), 43 participants (16.9%) had Spanish language preference.

**Table 1.** Enrollment and Follow-Up by Study Phase

Follow-up group	Screened families				
	Baseline, No. (%) (n = 555 [22.9%])	Intervention 1, No. (%) (n = 757 [31.2%])	Intervention 2, No. (%) (n = 681 [28.1%])	Sustainability, No. (%) (n = 431 [17.8%])	Total, No. (N = 2424)
Eligible	120 (21.6)	140 (18.5)	156 (22.9)	87 (20.2)	503
Ineligible^a^	433 (78.0)	612 (80.9)	525 (77.1)	344 (79.8)	1921^a^
Consented	87 (15.7)	84 (11.1)	84 (12.3)	71 (16.5)	326
Call completed among screened patients	68 (12.2)	65 (8.6)	63 (9.3)	60 (13.9)	256
Call completed among consented participants	68 (78.2)	65 (77.4)	63 (75.0)	60 (84.5)	256

^a^ Total number of families per reason for ineligibility (>1 reason possible per family): acute psychiatric presentation for 90 families, child age for 1199 families, language other than English or Spanish for 143 families, eligible but opted out for 71 families, child admitted for 422 families, no acetaminophen or ibuprofen for 329 families, eligible but missed (did not consent) for 118 families, and other for 223 families.

Across study phases, there were no significant differences in demographic characteristics, including child and parental age, language preference, race and ethnicity, and formal education. There were differences in the income distribution and health literacy of enrolled participants. For example, adequate health literacy among parents and guardians was found for 42 of 68 individuals (61.7%) at baseline, 43 of 65 individuals (66.2%) in intervention 1, 46 of 63 individuals (73.0%) in intervention 2, and 26 of 60 individuals at the sustainability phase (43.3%) (*P* = .02). Regarding participants’ prior experience with medical information and medication dosing, there was no difference in confidence with medical forms or mathematical calculations or experience with syringe dosing or study medications across study phases. (Table 2). Duration of the discharge process ranged by phase from a median (IQR) of 2.5 (2.0-4.0) minutes in the sustainability phase to 4.0 (2.0-5.0) minutes in the intervention 2 phase (eAppendix 5 in Supplement 1).

**Table 2.** Participant Demographics by Study Phase

Characteristic	Participants, No. (%) (N = 256)				*P* value^a^
	Baseline (n = 68 [26.6%])	Intervention 1 (n = 65 [25.4%])	Intervention 2 (n = 63 [24.6%])	Sustainability (n = 60 [23.4%])	
Age, median (IQR), y					
Child	3.0 (1.9-8.5)	2.0 (1.5-5.0)	4.0 (2.0-8.0)	2.4 (1.4-5.0)	.08
Parent or guardian	37 (31-42)	36 (32-40)	34 (28-40)	35.5 (33-41)	.46
Language preference for study					
English	58 (85.3)	55 (84.6)	53 (84.1)	46 (76.7)	.50
Spanish	10 (14.7)	9 (13.9)	10 (15.9)	14 (23.3)	
Missing or other	0	1 (1.5)	0	0	
Language preference for visit with doctors and nurses					
English	58 (85.3)	56 (86.2)	53 (84.1)	46 (76.7)	.48
Spanish	10 (14.7)	9 (13.8)	10 (15.9)	14 (23.3)	
Medication recommended by treating team					
Acetaminophen	4 (5.9)	3 (4.6)	4 (6.4)	6 (10.0)	.54
Ibuprofen	3 (4.4)	0	1 (1.6)	1 (1.7)	
Both	60 (88.2)	62 (95.4)	58 (92.1)	53 (88.3)	
Missing	1 (1.5)	0	0	0	
Child race and ethnicity					
Hispanic	46 (67.7)	27 (41.5)	31 (49.2)	28 (46.7)	.20
Non-Hispanic Black	1 (1.5)	4 (6.2)	4 (6.4)	5 (8.3)	
Non-Hispanic White	15 (22.1)	20 (30.8)	21 (33.3)	19 (31.7)	
Non-Hispanic other^b^	6 (8.8)	12 (18.5)	7 (11.1)	8 (13.3)	
Missing	0	2 (3.1)	0	0	
Parent or guardian race and ethnicity					
Hispanic	27 (39.7)	26 (40.0)	31 (49.2)	25 (41.7)	.86
Non-Hispanic Black	3 (4.4)	3 (4.6)	4 (6.4)	5 (8.3)	
Non-Hispanic White	31 (45.6)	26 (40.0)	23 (36.5)	21 (35.0)	
Non-Hispanic other^b^	7 (10.3)	9 (13.9)	5 (7.9)	9 (15.0)	
Missing	0	1 (1.5)	0	0	
Parent or guardian gender					
Male	20 (29.4)	19 (29.2)	10 (15.9)	7 (11.7)	.03
Female	48 (70.6)	46 (70.8)	53 (84.1)	53 (88.3)	
Confident with medical forms					
Extremely	45 (66.2)	38 (58.5)	39 (61.9)	38 (63.3)	.06
Quite a bit	18 (26.5)	20 (30.8)	12 (19.1)	16 (26.7)	
Somewhat	4 (5.9)	7 (10.8)	6 (9.5)	5 (8.3)	
A little bit	0	0	6 (9.5)	1 (1.7)	
Not at all	1 (1.5)	0	0	0	
Confident with mathematical calculations					
Extremely	38 (55.9)	28 (43.1)	34 (54.0)	28 (46.7)	.19
Quite a bit	16 (23.5)	26 (40.0)	19 (30.2)	15 (25.0)	
Somewhat	10 (14.7)	5 (7.7)	6 (9.5)	13 (21.7)	
A little bit	1 (1.5)	3 (4.6)	3 (4.7)	4 (6.7)	
Not at all	3 (4.4)	4 (4.6)	1 (1.6)	0	
Prior experience					
Syringe dosing	57 (83.8)	49 (75.4)	51 (81.0)	55 (91.7)	.17
Acetaminophen	66 (97.1)	64 (98.5)	63 (100.0)	58 (96.7)	.52
Ibuprofen	54 (79.4)	55 (84.6)	58 (92.1)	53 (88.3)	.35
Formal education					
Graduate degree	19 (27.9)	13 (20.0)	14 (22.2)	13 (21.7)	.77
Finished college	17 (25.0)	20 (30.8)	18 (28.6)	20 (33.3)	
Some college	9 (13.2)	17 (26.2)	11 (17.5)	8 (13.3)	
High school	19 (27.9)	13 (20.0)	17 (27.0)	15 (25.0)	
<Eighth grade	4 (5.9)	2 (3.1)	3 (4.8)	3 (5.0)	
Missing	0	0	0	1 (1.7)	
Annual income, $					
<20 000	3 (4.4)	1 (1.5)	3 (4.8)	5 (8.3)	.02
20 000-39 999	16 (23.5)	9 (13.9)	11 (17.5)	5 (8.3)	
40 000-59 999	4 (5.9)	4 (6.2)	7 (11.1)	9 (15.0)	
60 000-79 999	8 (11.8)	7 (10.8)	6 (9.5)	15 (25.0)	
80 000-99 999	5 (7.4)	6 (9.2)	4 (6.4)	3 (5.0)	
≥100 000	30 (44.1)	27 (41.5)	22 (34.9)	13 (21.7)	
Missing	2 (2.9)	11 (16.9)	10 (15.9)	10 (16.7)	
Adequate literacy by NVS score	42 (61.8)	43 (66.2)	46 (73.0)	26 (43.3)	.02

Abbreviation: NVS, Newest Vital Sign.

^a^ *P* values are from χ^2^ or Kruskal-Wallis tests as appropriate.

^b^ This includes individuals whose ethnicity was not Hispanic and whose race was not White or Black; other races included American Indian or Alaska Native, Asian, Native Hawaiian or Other Pacific Islander, other, and refuse.

Table 3 shows dosing outcomes by study phase, including medications used and errors by type using 3 definitions (any error, actual error, and potential use error). During the intervention 1 phase, 34 of 65 families (52.3%) demonstrated any error compared with 44 of 68 families (64.7%) at baseline. Rates of over- and underdosing error also decreased at intervention 1 compared with baseline (overdosing: 9 families [13.8%] vs 15 families [22.1%]; underdosing: 26 families [40.0%] vs 37 families [54.4%]).

**Table 3.** Outcomes by Study Phase

Outcome^a^	Participants, No. (%) (N = 256)			
	Baseline (n = 68)	Intervention 1 (n = 65)	Intervention 2 (n = 63)	Sustainability (n = 60)
**Dosing or frequency error**				
Any error	44 (64.7)	34 (52.3)	31 (49.2)	34 (56.7)
Actual error	25 (36.8)	24 (36.9)	17 (27.0)	23 (38.3)
Potential use error	43 (63.2)	34 (52.3)	30 (47.6)	34 (56.7)
**Overdosing error**				
Any error	15 (22.1)	9 (13.8)	5 (7.9)	7 (11.7)
Actual error	7 (10.3)	6 (9.2)	3 (4.8)	4 (6.7)
Potential use error	15 (22.1)	9 (13.8)	5 (7.9)	7 (11.7)
**Underdosing error**				
Any error	37 (54.4)	26 (40.0)	29 (46.0)	31 (51.7)
Actual error	21 (30.9)	19 (29.2)	16 (25.4)	21 (35.0)
Potential use error	36 (52.9)	26 (40.0)	28 (44.4)	31 (51.7)

^a^ Each row is an independent result given that families could have more than 1 type of error and so appear in more than 1 row. Column percentages are shown but do not sum to 100% because groups overlap.

### Qualitative: Washout Period

A total of 11 interviews were conducted with ED clinicians, including 3 nurses (RN degrees), 3 attending physicians (MD degrees), and 5 resident physicians (MD degrees). Clinicians had a mean (SD) 4.7 (3.3) years of ED experience in their current role. Interviews were completed over 2 months. Overall, 7 themes were generated from our data as mapped to DSF elements: 3 themes related to intervention, 2 themes related to practice setting, and 2 themes related to the ecological system (eAppendix 4 and 6 in Supplement 1).

#### Care Improvement

Physician and nursing participants reported that they felt strongly that the intervention improved care and allowed them to better address parental concerns; they specifically emphasized improved handouts. A nurse said, “I think it’s great to send home…the dosing chart, because they feel like a lot of parents are nervous whether to give too much, and then sometimes they give too little and that causes them to bring the child in…because it’s not working.” Other participants emphasized how the study had improved their own discharge teaching and that they appreciated the opportunity to confirm understanding (eAppendix 4 in Supplement 1).

#### Modifications to Training and Implementation

Physicians and nurses emphasized the importance of individual training compared with a video. They suggested changes to specific elements of the intervention (eAppendix 1 and 4 in Supplement 1).

#### Appropriate Role Group for Intervention Delivery

Physicians commented that they felt the intervention was best delivered by nursing colleagues. A physician said, “This is kind of a cop-out, but I really do think you’re going to have a lot better uptake if the nurses do it when they give patients the discharge paperwork because they’re the ones who are actually physically giving them the discharge paperwork.”

In contrast, nursing respondents emphasized that they were not responsible for writing instructions and so were dependent on receiving the written instructions from physicians or separately obtaining the simplified dosing handout. Nurses said they felt the intervention would benefit from better reminders for physicians to insert intervention instructions (eAppendix 4 in Supplement 1).

#### Practice Setting

##### Importance of a Local Champion

Physicians and nurses discussed the importance of learning about the intervention from a trusted colleague in a meeting or on a clinical shift. They said this was more useful than emails or visual reminders for continued uptake of the intervention (eAppendix 4 in Supplement 1).

##### Timing Constraints for Clinicians and Parents and Guardians

The most frequently discussed theme was time constraints. A physician described time pressure from patient volume as “I think it just takes time, and it’s an extra step. And when there’s people waiting in the ED, we need every minute we can get.” Nurses highlighted time constraint concerns on the family side, particularly considering how the time of the visit may affect parents and guardians wanting to leave without intervention teaching.

#### Ecological System

##### External Barriers to Medication Safety

Clinicians discussed how the intervention training made them more aware of barriers that parents and guardians may face attempting to dose medications safely at home and a reminder that many over-the-counter products are not supplied with appropriate dosing instruments (such as oral syringes). A nurse said, “It’s giving them something that they can measure it with because…they don’t have those at home, or with the over-the-counter products, they don’t supply that.”

##### Communication Challenges

Clinicians described discharging parents and guardians who reported complete comprehension but left the clinician concerned that more teaching might be needed. For those individuals and individuals with limited English proficiency or health literacy, clinicians described the intervention as being particularly useful. A nurse said, “I think it’s really nice to have the tool because, especially sometimes with language barrier or anything like that, it’s nice to be able to have something you can show them.”

#### Implications: Changes for Intervention 2

Qualitative interviews led to the inclusion of improved visual reminders and just-in-time training for the intervention, the inclusion of a pictorial syringe dosing diagram on handouts, additional real-time support around intervention delivery and training in the ED, and increased emphasis on providing a dosing syringe. Additional modifications included changes to the language on the handout to increase clarity: ensuring that brand and generic names were provided for medications, dosing intervals were clearly described, and the safety of coadministration of acetaminophen and ibuprofen was explicitly mentioned. Perhaps the biggest modification of the intervention was the transition from a focus on teach-back, which was perceived by clinicians to be optimal but time prohibitive, to a focus on dose demonstration, which was seen as more feasible (eAppendix 1 in Supplement 1).

### Quantitative: Intervention 2 and Sustainability

During intervention 2, 31 of 63 families (49.2%) demonstrated any error, compared with 52.3% in intervention 1; rates of overdosing error decreased compared with intervention 1 (5 families [7.9%] vs 13.8%), although the rate of underdosing error increased (29 families [46.0%] vs 40.0%). Rates of error were higher in the sustainability phase (34 of 60 families [56.7%]), although still improved from the baseline (64.7%).

Overall, the odds of any error in the combined intervention phases (intervention 1 and 2) were decreased compared with the baseline even after adjustment for language and health literacy (adjusted odds ratio, 0.52 [95% CI, 0.28-0.97). Table 4 shows unadjusted and adjusted models for all definitions of error. Examining secondary process outcomes (eAppendix 5 in Supplement 1), there was no significant increase in the time of the discharge process, but the use of study interventions increased during intervention periods and was moderately persistent during the sustainability phase.

**Table 4.** Odds of Dosing Errors in Combined Training Phases vs Baseline

Outcome	OR (95% CI)^a^	
	Unadjusted	Adjusted^b^
**Dosing or frequency error**		
Any error	0.54 (0.29-0.99)	0.52 (0.28-0.97)
Actual error	0.75 (0.40-1.40)	0.74 (0.39-1.38)
Potential use error	0.55 (0.30-1.01)	0.53 (0.29-0.98)
**Overdosing error**		
Any error	0.38 (0.16-0.86)	0.38 (0.17-0.88)
Actual error	0.52 (0.17-1.54)	0.57 (0.19-1.72)
Potential use error	0.38 (0.16-0.86)	0.38 (0.17-0.88)
**Underdosing error**		
Any error	0.62 (0.34-1.12)	0.60 (0.33-1.10)
Actual error	0.80 (0.42-1.54)	0.77 (0.40-1.49)
Potential use error	0.63 (0.35-1.15)	0.61 (0.33-1.11)

Abbreviation: OR, odds ratio.

^a^ The reference group is the baseline phase for all outcomes.

^b^ Adjusted for language choice (English vs Spanish) and health literacy (as measured by the Newest Vital Sign).

## Discussion

In this mixed-methods study, training and implementation support were associated with decreased parental risk of dosing errors at home after ED discharge. Our findings suggest that the MEDS intervention may be successfully delivered by clinicians without requiring a marked increase in time of discharge process. Rates of intervention use were higher and rates of error lower during intervention phases compared with the passive sustainability phase, but improvement was sustained compared with baseline.

These findings may strengthen the evidence base for the MEDS intervention^17^ by adding information about clinician perspectives and strategies for sustainable implementation (eAppendix 6 in Supplement 1). Clinicians recognized multiple areas for improvement in ED discharge practices, similar to those described by patients and parents and guardians.^29^ Improved practices for medication discharge teaching, including visual aids^24^ and standardization of dosing devices^30^ have been recommended for many years but remain challenging to implement in practice. Continued attempts to develop simplified, brief interventions, with careful attention to how best to maximize the fit between interventions and practice settings, may help to improve implementation of these strategies. Recognizing that the ED is only 1 example of a setting in which complex instructions are given to parents and guardians in a time-limited setting, these lessons may also have broad applicability in primary care, in-patient discharge, and postprocedural settings.

Nurses viewed physicians as responsible for the creation of instructions, and physicians described nurses as being responsible for the delivery of instructions. Although preprinted handouts were available such that nurses could retrieve them without physician involvement, we repeatedly heard the expectation that instructions should be placed by physicians into the medical record prior to the nurse discharge teaching. Next steps could include automatic insertion of materials into discharge instructions. Given that the role of clinical pharmacists is increasingly recognized as integral to high-quality ED care,^31^ opportunities exist to use their expertise in medication counseling. Additionally, pharmacists, physicians, and nurses could provide more education earlier in the visit rather than waiting until discharge.

### Limitations

This study has several limitations. It was conducted at a single academic ED and enrolled a convenience sample. Given the modest sample size, we could adjust only for language and literacy as potential confounders. The study was not designed to estimate medication-related harm given that we assessed a hypothetical dosing question and actual error, but it provides evidence that the MEDS intervention may be associated with improved understanding of safe dosing. From a qualitative perspective, we interviewed clinicians over the phone, limiting our ability to assess nonverbal cues. Additionally, our work may be subject to social desirability bias in which interview participants did not want to criticize provided services to someone perceived as being a representative of the intervention or study team, and the qualitative work during the washout period may have reminded clinicians of the intervention. However, only a small number of clinicians were involved in interviews. In addition, while we describe the variation in themes by clinician group, this is not a prevalence sample and is designed only to describe the range of potential themes.

## Conclusions

This mixed-methods study found that both MEDS intervention phases were associated with decreased risk of error compared with baseline and that some improvement was sustained without active intervention. Participants said they felt the intervention helped them provide better care and identified improvements to the intervention and areas to address in subsequent implementation efforts. These data emphasize the importance of providing simple instructions for clinicians to use, giving specific teaching to clinicians about how and what to teach, and having a local champion and continued training. Overall, these findings suggest that the MEDS intervention may be successfully taught to clinicians, delivered within routine ED practice, and associated with decreased risk of error for families after discharge.

## Notes

Supplement 1. **eAppendix 1.** Framework for Reporting Adaptations and Modifications-Expanded (FRAME) Summary **eAppendix 2.** Interview Guide **eAppendix 3.** Coding Tree **eAppendix 4.** Themes and Representative Quotes Mapped to Elements From Dynamic Sustainability Framework **eAppendix 5.** Discharge Process by Study Phase **eAppendix 6.** Conceptual Model of Themes as Adapted From Dynamic Sustainability Framework

Supplement 2.
Data Sharing Statement
