# Supplementary material for: Analysis of a Medication Safety Intervention in the Pediatric Emergency Department
Source: JAMA Netw Open. 2024 Jan 12;7(1):e2351629. doi: 10.1001/jamanetworkopen.2023.51629 (PMC10787317; doi:10.1001/jamanetworkopen.2023.51629)
Supplement: Supplement 1. — eAppendix 1. Framework for Reporting Adaptations and Modifications-Expanded (FRAME) Summary eAppendix 2. Interview Guide eAppendix 3. Coding Tree eAppendix 4. Themes and Representative Quotes Mapped to Elements From Dynamic Sustainability Framework eAppendix 5. Discharge Process by Study Phase eAppendix 6. Conceptual Model of Themes as Adapted From Dynamic Sustainability Framework [file jamanetwopen-e2351629-s001.pdf]

Supplemental Online Content

Samuels-Kalow ME, Tassone R, Manning W, et al. Analysis of a medication safety intervention in the pediatric emergency department. *JAMA Netw Open*. 2024;7(1):e2351629. doi:10.1001/jamanetworkopen.2023.51629

eAppendix 1. Framework for Reporting Adaptations and Modifications-Expanded (FRAME) Summary

eAppendix 2. Interview Guide

eAppendix 3. Coding Tree

eAppendix 4. Themes and Representative Quotes Mapped to Elements From Dynamic Sustainability Framework

eAppendix 5. Discharge Process by Study Phase

eAppendix 6. Conceptual Model of Themes as Adapted From Dynamic Sustainability Framework

This supplemental material has been provided by the authors to give readers additional information about their work.

© 2024 Samuels-Kalow ME et al. *JAMA Network Open.*

Appendix 1: FRAME Summary

The FRAME includes 8 aspects: when and how the modification was made, whether it was planned or unplanned, who determined the modification should be made, what was modified, at what level of delivery the modification was made, the type or nature of the modification, the extent to which the modification was fidelity-consistent, and the reasons for modification including the goals and contextual factors.

**Initial implementation strategies**								
Video Training and Initial Informational Email (available from authors by request)								
EMR Handouts (available from authors by request)								
Handout Binder								
Reminder Stickers on Computer								
RA Support								
**Phase 2 Modifications**	**When modification made?**	**Planned or unplanned?**	**Who determined?**	**What was modified?**	**What level of delivery**	**Nature of modification?**	**Fidelity consistent?**	**Reasons for?**
Syringe basket	Phase 2	Planned*	Study team	Location of materials	Unit level	Adding a basket of oral syringes to an easy to access portion of the nurse workspace	Yes	Reduce barrier of needing to find equipment
Handout: pounds	Phase 2	Planned	Study team	Handout	Organization	Added weight in pounds	Yes	Not all parents may know child's weight in kilograms
Handout: oral syringe	Phase 2	Planned	Study team	Handout	Organization	Added picture of oral syringe	Yes	Pictographs may improve understanding
Handout: color change	Phase 2	Planned	Study team	Handout	Organization	Change red/yellow colors to blue yellow and green	Yes	Clinician team thought this would be easier to read
Handout: Weight phrase	Phase 2	Planned	Study team	Handout	Organization	Add growing out of weight range phrase	Yes	Clinician's concern that child may

© 2024 Samuels-Kalow ME et al. *JAMA Network Open.*

								outgrow medication dose
Handout: syringe	Phase 2	Planned	Study team	Handout	Organization	Add distribute syringe phrase	Yes	Additional reminder for dosing syringe distribution
Handout: Motrin pathway	Phase 2	Planned	Study team	Handout	Organization	Consolidate final bubble on Motrin dual pathway	Yes	Improve readability of handouts

**Proposed modifications that were not implemented**	**Justification**
Morning Conference training	Scheduling constraints (residents rotating in and out of the ED frequently, so unclear how many would be reached by a given conference and then have an ED rotation)
Handout: Add info on Staggering	Differences of opinion among ED clinical staff on appropriateness of staggering (alternating) medications v. using a single medication
Handout: Add all Brands	Concerns about increasing confusion and perception of brand endorsement; remained with generic names on handouts.

*****Planned "because it was determined through a systematic process rather than through improvisation" [1]

© 2024 Samuels-Kalow ME et al. *JAMA Network Open.*

Appendix 2: Interview Guide

Provider Guide for Qualitative Interviews

Provider Initials:
Date:

Value Neutral Transitions:

- Thank you
- Could you expand on that?
- How would that work?
- I see
- mmm
- Okay
- Is there anything more you'd like to add?

Introduction and Consent:

1) Have consent form ready.
2) Thank them for their time.
3) Before we get started, I want to let you know we are using a recorded phone line for the purposes of collecting qualitative data from these interviews. **Is that ok with you?**
4) Also, before we get started, I just wanted to check-in and see if you had any questions about the MEDS study or fact sheet that was sent to you via email?
5) *"To get started, I first would like to know more about you…"*
   a. What is your current role in the ED and how much experience do you have in this role?
   b. What languages do you speak?

Exploring Provider Understanding and Learning:

1) What is your understanding of the MEDS intervention steps? → **If they report no/little understanding add…** *Would you like a reminder of the three steps and goals of the study?*
2) Where did you first learn about the MEDS intervention?
3) Have you learned about the MEDS intervention anywhere else?
4) **If not mentioned,** did you watch/receive the instruction video sent via email last November?
   a. **If yes above,** what were your thoughts on the instruction video?
   b. **If yes above,** was there anything particularly helpful about the video?
   c. **If yes above,** was there anything that could be made better about the video?
5) **If not mentioned,** have you worked with any of our research assistants in the ED to learn about and implement the MEDS intervention?
   a. **If yes above,** what was that experience like for you?
   b. **If yes above,** is there anything the research assistants are doing that is particularly helpful for learning and implementing the MEDS intervention?

© 2024 Samuels-Kalow ME et al. *JAMA Network Open.*

c. **If yes above,** is there anything the research assistants can do better to help support providers like yourself understand and use the MEDS intervention?

6) **If mentioned any learning opportunities add…** *"Through all the exposures and learning mechanisms you just mentioned,"* Do you have any thoughts on teaching providers about the MEDS intervention?

a. Was there anything that made it easier for you to learn the intervention steps?

b. Was there anything that made it harder for you to learn about the intervention?

7) Overall, how do you think we can best teach providers like yourself about the MEDS intervention? *Specific suggestions for follow-up:*

a. Do you have any ideas for new ways for outreach to providers?

b. Do you have suggestions for new methods to train providers?

c. What aspects of the MEDS intervention do you think are most crucial to provide training for?

Feedback on Intervention Materials:

1) What are your thoughts on the new discharge materials? *Specific suggestions for follow-up:*

a. Thoughts on handouts?

b. Thoughts on provision of syringe and information to parents?

c. Thoughts on teach-back?

2) **If not mentioned,** is there anything you feel we should add to the materials?

3) **If not mentioned,** is there anything you feel should be changed about the current materials?

Exploring Provider Experience with Intervention:

4) Have you discharged anyone using the new process *(ANY of the three components)*?

a. **If yes above,** how did it go?

b. **If not mentioned,** can you tell me about a positive experience you have had using the intervention?

© 2024 Samuels-Kalow ME et al. *JAMA Network Open.*

i) **If yes above,** why do you think it went well?

c. **If not mentioned,** can you tell me about a negative experience you have had using the intervention?

i) **If yes above,** why do you think it did not go well?

5) **If they report never using the MEDS intervention,** ask them to imagine the scenario and try to obtain hypothetical responses.

6) What do you think could be done to improve the intervention delivery process itself for providers like you? → **If they need clarification add…** *"In this case, I am less focused on the materials or training environment and more focused on the real-life experience of walking into a patient room and using the MEDS process."*

Closing:

1) Is there anything else you would like to add that you feel we have not discussed or left out?

2) Remind them of the gift card and ask for their mailing address to get it to them.

3) Thank them again for their time.

4) Make sure consent form is filled out.

© 2024 Samuels-Kalow ME et al. *JAMA Network Open.*

Appendix 3: Coding Tree

1. Training
    a. Barriers
    b. Facilitators
2. Study intervention
    a. Barriers
    b. Facilitators
3. Prior discharge experiences
    a. Positive
    b. Negative
4. New ideas
5. Quotes to use

© 2024 Samuels-Kalow ME et al. *JAMA Network Open.*

Appendix 4: Themes and representative quotes mapped to elements from the dynamic sustainability framework[3]

Element	Theme	Physician	RN
**Intervention**	Care improvement	Yeah and I think for me, some of the things that were compelling was definitely knowing some of the background literature on why this is important and the fact that patients leave and don't remember anything that we tell them in a discharge process. So having things written down is important. And then also the teach-back method in particular has been pretty well proven as being efficacious. And so I think knowing that- everyone wants to do right by their patients, right, and so knowing that the things you're taking the time to do are effective, I think is really valuable.<br><br>Yeah, because the parents seemed like relieved. And in fact, it's just something that parents a lot of the time will ask us for, like, "Oh, could you tell me the dosage for ibuprofen or Tylenol that I should be giving my child?"<br><br>Everyone should get the handout regardless of what they're there for	I love the handout because it's so great, because it actually puts the child's weight on there … it's so much easier to be able for parents to see okay, here's my exact child's weight, and I can just put it right into the chart, as opposed to when you're reading on the back of instructions on the box, it's not as easy to visualize, and you don't have your child's up-to-date current weight from that day. So I think that's really helpful.<br><br>It made me think more about how often we just say to take Tylenol and ibuprofen at home, and then we don't really go over it.<br><br>I think it's great because I think that it will definitely solidify that they know what they're doing and that they can demonstrate it. And then that way when they get home, they're like, "Oh, I've already done this, and I know." And it also gives the provider the opportunity to kind of stop and say…where are we coming up short in kind of showing this to them?" …And then we can kind of focus on that area whether they're not understanding how to use this oral syringe or they're not understanding the chart
	Modifications to training and implementation	Yeah. I don't know how practical this is, but most of the time-- or whether it's already being implemented. But most of the time when we're discharging someone with Tylenol and ibuprofen, we've already given them some in the emergency department as part of their care because they had a fever or pain. So it sort of feels like they could do the teaching at the time when they get their first	I think maybe specific examples and just maybe someone explaining what the best way to go over the discharges, and just going step by step through like, "Okay, here's the chart. Here's how you should explain to the parents, 'Oh, this is the weight. This is the dose. This is the syringe you would use.'" And then having them be like, "Okay. And at this point, you should give the syringe to

© 2024 Samuels-Kalow ME et al. *JAMA Network Open.*

		dose so that it doesn't have to be reviewed later. I think, practically speaking, that's probably hard to implement, but since the nurse is going to have to go in there and administer Tylenol and Motrin at some point during the visit, that could be the time to show them drawing it up and administration to the family<br><br>I think if parents could also see the weight in pounds as opposed to just kilograms.… that would be helpful, because parents usually don't know their kids weight in kilograms. And then since there's a good amount of white space, if there could be a picture of a syringe and show it being filled to what the right level would be, what 3.75 milliliters looks like on a syringe, I think that'd be helpful too.<br><br>(re video training): I wouldn't be able to remember the specific details offhand at this point, but it did give me a quick plug into the fact that there is this program available	the parents and then show them." Kind of like a step-by-step kind of walkthrough of more as the nurses' role, that's where we come in to explain it. … have a step-by-step to go through in your head of, "Okay, I need to hit these points before I send them home."
	Divergent opinions about appropriate role group for intervention delivery	We just sometimes don't have the time. I know that attending because especially the PEDs trained attendings are really good at it, and all they do is see kids, right, so it's easier for them to do it than it's for the residents who are mostly seeing adults<br><br>I think the most important one to educate the providers about is which handout to attach to the discharge instructions so we give them the right thing because that's sort of like the piece that I think we can easily implement, is just clicking on the right handout to make sure that the parents get it. I think the teaching piece at the MD level, that probably is not going to be in the workflow very easily since the	So I think where maybe the disconnect in terms of nursing and the doctors and MDs is that we get the instructions, and so sometimes we don't write them, so we print them out. And so sometimes, if the chart's not included in there, it's a little like, "Okay. I am just reading-- I'm just going through the discharge that they wrote." And so I think maybe just prompting the providers to remember to put those charts in the discharge. And I think the dot phrases will be great. I think that will be a huge step because we're the ones at the bedside explaining, but we don't write the instructions, so I think it will be nice to have a little bit more of a reminder for them to make sure to include that stuff, writing instructions.

© 2024 Samuels-Kalow ME et al. *JAMA Network Open.*

		nurses are primarily the ones who discuss the discharge.	I actually asked one day if one of the doctors could add one on because someone had come in because they were under dosing on the ibuprofen and Tylenol
**Practice setting**	Importance of a local champion	Yeah, I think we tend to do best with kind of like peer to peer-- basically, one good way, although not everybody comes to faculty meeting, is just to have a faculty member who's interested in this intervention or whether from a study perspective or from quality improvement perspective, present that to their peers so that-- it tends to go over well when it's sort of a colleague to colleague. I think that works well. And then, of course, we forget about it like a month later. And I would say email doesn't work so great just because of the huge volume of emails that everybody gets these days. So I think having some time in the faculty meeting from a peer or intermittent reminders through the year, and that format works pretty well.	So a couple times per shift, if somebody just rounds through the area, and it's like, "Hey. We're doing this new thing. Let's chat about it for a hot second."
	Timing constraints—both for clinicians and parents	What's time-consuming is the cumulative nature of it, right. If you're doing it for every single patient that you're discharging, which we probably should be doing, then the cumulative nature becomes-- then it becomes a burden and time-consuming. So figuring out an efficient way to do it for every single patient that needs it, which is essentially every single patient, is the best way.<br><br>I think that's great. I think a lot of people have trouble with dosing, and I've seen a lot of patients who have been given the wrong dose because they don't know how to use a syringe correctly. So I think actually demonstrating and having them practice is really important. It's just something that we usually don't have time for.	Sometimes only just because, again, it depends on how busy we are that day, and how long they've been there, and how eager they are to leave. And so I think when people are fatigued from being in the ER for so long, they're not as willing or able to take in a lot of information at that time. So I think maybe doing, like I said, during the day. If you do give the meds, have an opportunity to do it then. People might be a little bit more willing to listen and absorb.<br><br>I really like the idea of it, and I really like that, hopefully, it will improve medicating children at home and getting them feeling better and staying out of ED, which are all things that I heartily approve of. I think we just really need to be able

© 2024 Samuels-Kalow ME et al. *JAMA Network Open.*

			to implement it and implement it well. And I think my biggest concern is that we just don't have the-- which is nothing that, unfortunately, you guys can do, but the time and the staff to really sit down and go through it with patients and families. So the more streamlined it can be, I think, the better.<br><br>And it's not all patients, but some patients are just very fatigued from being there, and so they're kind of just over it at that point
**Ecological system**	External barriers to medication safety	I think that's great. Yeah. I think the teach back is important because I feel like most of the time what we do is we don't have time to teach back. And we just assume that like, "Well, you're a parent. You probably know. You've been at it for a couple of years. You probably know how to dose Tylenol and ibuprofen at this point. And I'm just telling you what the dose is, and you can go and keep doing it." So I like to teach back because it actually verifies that the parents understand what they're doing<br><br>I guess the video just reminded me of the importance of it, that families don't necessarily understand dosing in concentrations like we may	I think that-- hopefully, it will prevent repeat visits to the ED. I think, unfortunately, a lot of people use ED as their primary care, or they just really are at a loss when their child just needs Tylenol or Motrin. So hopefully with the teaching, they'll understand they don't need to come in for just that….And a lot of times parents don't know the correct dose.<br><br>I think it's really great and it's really important, and maybe people undervalue it because it's like, "Oh, it's just over-the-counter meds," but they're really important in reducing the amount of ED visits that we get and keeping the kids out of the hospital and things like that.
	Communication challenges faced by patients and parents	I think knowing that there's the demonstration is helpful for us because I think that is pretty valuable, especially for patients with limited English proficiency.	I love using the handout. …even with … a language barrier or whatever it is-- if I have a handout and I can highlight and say, "5 MLs," and show, "This is how much you should be giving," it seems like they understand that a lot better than me just talking at them and saying, "This is how much you should be giving." So it makes me feel

© 2024 Samuels-Kalow ME et al. *JAMA Network Open.*

			better sending them home when I can hand them a chart that says, "This is how much you need to give." And I feel like it makes the parents a little bit more comfortable also.<br><br>The ED is stressful, and you're throwing a lot of info at parents.. So, I think a lot of them smile and nod, and they're like, "Yep. Yep. I totally get it. I totally get it." And you're like, "Um, okay. I don't know that I completely believe you, but okay

© 2024 Samuels-Kalow ME et al. *JAMA Network Open.*

Appendix 5: Discharge process by study phase (n=256).

Discharge characteristic	Baseline	Intervention 1	Intervention 2	Sustainability	p*
Provider type†					0.62
Physician (attending)	0 (0)	0 (0)	0 (0)	0 (0)	
Physician (resident)	3 (4)	4 (6)	5 (8)	6 (10)	
Registered nurse	64 (94)	61 (94)	57 (90)	53 (88)	
Medical student	1 (1)	0 (0)	0 (0)	0 (0)	
Missing	0 (0)	0 (0)	1 (2)	1 (2)	
Duration of discharge process in minutes, median (IQR)	3 (2-5)	3 (2-5)	4 (2-5)	2.5 (2-4)	0.05
Medication instructions given					0.54
Tylenol	4 (6)	3 (5)	4 (6)	6 (10)	
Motrin	3 (4)	0 (0)	1 (2)	1 (2)	
Both	60 (88)	62 (95)	578 (92)	53 (88)	
Missing	1 (1)	0 (0)	0 (0)	0 (0)	
Language used/interpretation					0.01
English	59 (87)	59 (91)	58 (92)	53 (88)	
Hospital translator used	0 (0)	0 (0)	1 (2)	5 (8)	
Family member translator used	3 (4)	0 (0)	0 (0)	0 (0)	
Phone interpreter used	4 (6)	5 (8)	4 (6)	1 (2)	
Provider spoke Spanish	0 (0)	0 (0)	0 (0)	1 (2)	
Other/missing	2 (3)	1 (2)	0 (0)	0 (0)	
Usage and demonstration of Tylenol (n=249)					
Medication demonstrated	0 (0)	5 (8)	34 (56)	1 (2)	<0.001
Use described (any teaching)	14 (22)	45 (69)	58 (94)	3 (5)	<0.001
Use described (dose/unit/frequency)	23 (36)	46 (71)	56 (90)	17 (28)	<0.001
Use not discussed	25 (39)	5 (8)	1 (2)	40 (68)	<0.001
Medication provided to parent	0 (0)	0 (0)	0 (0)	0 (0)	n/a
Only prescription given to parent	0 (0)	0 (0)	0 (0)	4 (7)	0.004
Not applicable (not using medication)	2 (3)	2 (3)	1 (2)	7 (12)	0.04
Usage and demonstration of Motrin (n=237)					
Medication demonstrated	0 (0)	6 (10)	34 (58)	0 (0)	<0.001
Use described (any teaching)	16 (25)	41 (66)	55 (93)	3 (6)	<0.001
Use described (dose/unit/frequency)	17 (27)	43 (69)	53 (90)	14 (26)	<0.001
Use not discussed	26 (41)	6 (10)	1 (2)	37 (69)	<0.001
Medication provided to parent	0 (0)	0 (0)	0 (0)	0 (0)	n/a
Only prescription given to parent	1 (2)	0 (0)	0 (0)	1 (2)	0.55
Not applicable (not using medication)	4 (6)	2 (3)	1 (2)	8 (15)	0.02
Parent had opportunity to ask questions	62 (91)	62 (95)	60 (95)	58 (97)	0.20

© 2024 Samuels-Kalow ME et al. *JAMA Network Open.*

Location of paper discharge instructions					0.03
No instructions handed to parent	0 (0)	3 (5)	0 (0)	0 (0)	
Parent given instructions but left behind	2 (3)	0 (0)	0 (0)	0 (0)	
Parent given instruction and left with them	66 (97)	62 (95)	63 (100)	60 (100)	
Use of demonstration/dosing sheets					
Used study teaching sheets	0 (0)	60 (92)	59 (94)	22 (37)	<0.001
Demonstrated dose	0 (0)	17 (26)	38 (60)	1 (2)	<0.001
Performed teach-back	0 (0)	4 (6)	8 (13)	0 (0)	0.001
Provided syringe for discharge	1 (1)	60 (92)	56 (89)	0 (0)	<0.001
Did none of the above	64 (94)	3 (5)	3 (5)	37 (62)	<0.001
Demonstration					
No demonstration provided	0 (0)	10 (15)	21 (33)	57 (95)	<0.001
Demonstration with dosing syringe only	0 (0)	19 (29)	34 (54)	0 (0)	<0.001
Demonstration with dosing syringe & study bottles	0 (0)	0 (0)	0 (0)	1 (2)	0.35
Other	0 (0)	0 (0)	3 (5)	2 (3)	0.12
Teach back (all 1 cycle)	0 (0)	4 (6)	4 (6)	0 (0)	0.04

Abbreviations: IQR, interquartile range (reported as quartile 1-quartile 3).
*P-values from Chi-square or Kruskal-Wallis tests, as appropriate.
†Missing 2 (1 in training 2, 1 in sustainability).

Appendix References

1. Wiltsey Stirman S, Baumann AA, Miller CJ. The FRAME: an expanded framework for reporting adaptations and modifications to evidence-based interventions. *Implement Sci* 2019; **14**(1): 58.
2. Tong A, Sainsbury P, Craig J. Consolidated criteria for reporting qualitative research (COREQ): a 32-item checklist for interviews and focus groups. *International Journal for Quality in Health Care* 2007; **19**(6): 349-57.
3. Chambers DA, Glasgow RE, Stange KC. The dynamic sustainability framework: addressing the paradox of sustainment amid ongoing change. *Implement Sci* 2013; **8**: 117.

© 2024 Samuels-Kalow ME et al. *JAMA Network Open.*

Appendix 6: Conceptual model of themes as adapted from the dynamic sustainability framework [3]

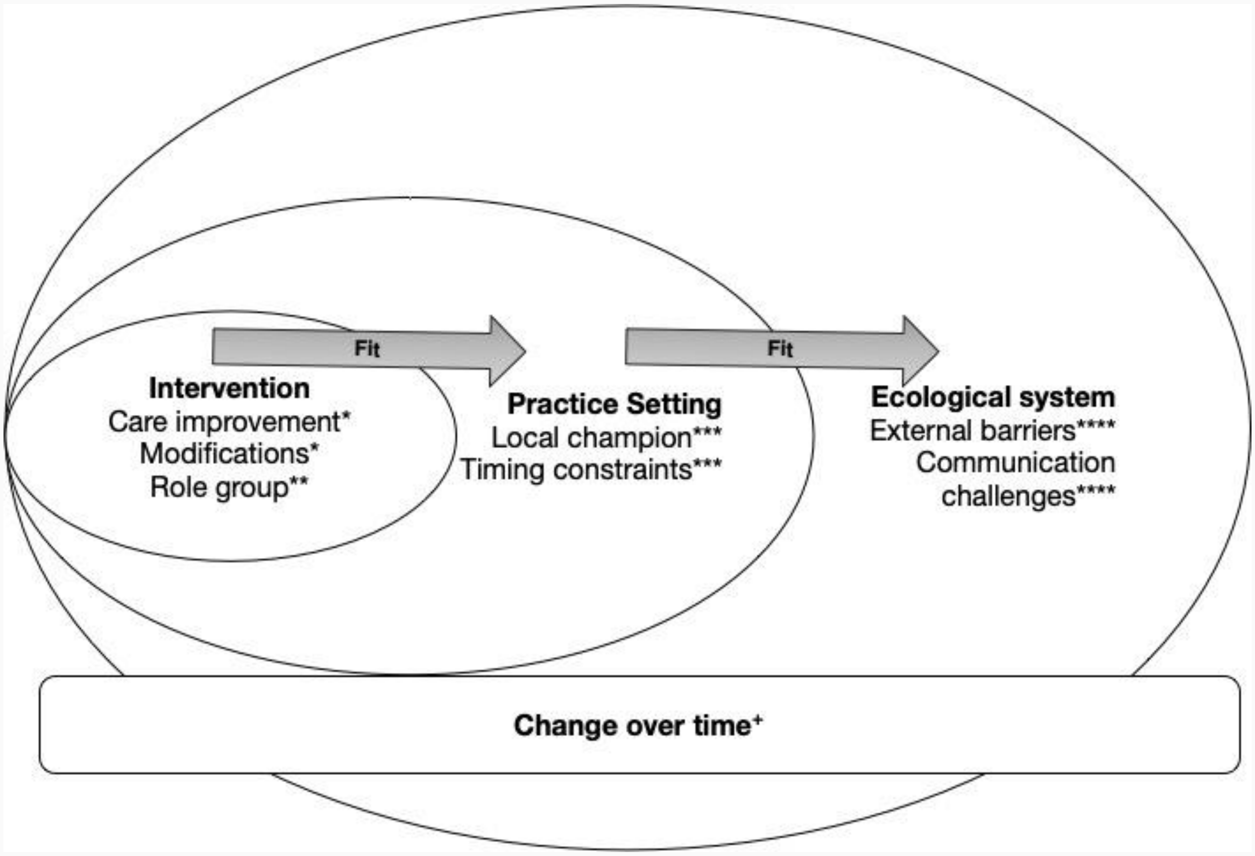

*Participants described overall positive feelings toward the intervention, and felt that it helped them provide higher quality care to patients and families, but also provided suggestions for intervention and implementation improvement.
**Physicians and nurses identified implementation challenges around role group assignments, as the existing implementation approach required tasks that, although they could be completed by either role group, were generally considered to be either physician tasks (instruction insertion) or nurse tasks (discharge instruction teaching).
***Regarding the practice setting, clinicians discussed the importance of a local champion and strongly emphasized the constraints of time pressures on their ability to provide the intervention consistently.
****Participants reported the intervention made them more aware of limitations within the ecological system—both external barriers to medication safety around existing information provided to parents (e.g. on over-the-counter product boxes) and dosing implements (cups provided with over-the-counter medications)—and communication barriers such as language and health literacy.
+ An important component of the DSF model is change over time, and several clinicians described how the intervention training changed their perspective and practice during the study period. As another component of change over time, the recommended training improvements were incorporated into the intervention implementation strategy, and included in the second phase.

© 2024 Samuels-Kalow ME et al. *JAMA Network Open.*
