# Supplementary material for: Analysis of a Medication Safety Intervention in the Pediatric Emergency Department
Source: JAMA Netw Open. 2024 Jan 12;7(1):e2351629. doi: 10.1001/jamanetworkopen.2023.51629 (PMC10787317; doi:10.1001/jamanetworkopen.2023.51629)
Supplement: Supplement 2. — Data Sharing Statement [file jamanetwopen-e2351629-s002.pdf]

Data Sharing Statement

Samuels-Kalow. Analysis of a Medication Safety Intervention in the Pediatric Emergency Department. *JAMA Netw Open*. Published online January 12, 2024. doi:10.1001/jamanetworkopen.2023.51629

Data
Data available: No
